# Phylogeographical Structure in Mitochondrial DNA of Legume Pod Borer (*Maruca vitrata*) Population in Tropical Asia and Sub-Saharan Africa

**DOI:** 10.1371/journal.pone.0124057

**Published:** 2015-04-20

**Authors:** Malini Periasamy, Roland Schafleitner, Krishnan Muthukalingan, Srinivasan Ramasamy

**Affiliations:** 1 Asian Vegetable Research and Development Center (AVRDC)—The World Vegetable Center, PO Box 42, Shanhua, Tainan 74199, Taiwan; 2 Department of Environmental Biotechnology, Bharathidasan University, Tiruchirappalli 620024, Tamil Nadu, India; Field Museum of Natural History, UNITED STATES

## Abstract

This study was undertaken to assess the genetic diversity and host plant races of *M*. *vitrata* population in South and Southeast Asia and sub-Saharan Africa. The cytochrome c oxidase subunit 1 (*cox1*) gene was used to understand the phylogenetic relationship of geographically different *M*. *vitrata* population, but previous studies did not include population from Southeast Asia, the probable center of origin for *Maruca*, and from east Africa. Extensive sampling was done from different host plant species in target countries. Reference populations from Oceania and Latin America were used. An amplicon of 658 bp was produced by polymerase chain reaction, and 64 haplotypes were identified in 686 *M*. *vitrata* individuals. Phylogenetic analysis showed no difference among the *M*. *vitrata* population from different host plants. However, the results suggested that *M*. *vitrata* has formed two putative subspecies (which cannot be differentiated based on morphological characters) in Asia and sub-Saharan Africa, as indicated by the high pairwise *F_ST_* values (0.44–0.85). The extremely high *F_ST_* values (≥0.93) of *Maruca* population in Latin America and Oceania compared to Asian and African population seem to indicate a different species. On the continental or larger geographical region basis, the genetic differentiation is significantly correlated with the geographical distance. In addition, two putative species of *Maruca*, including *M*. *vitrata* occur in Australia, Indonesia and Papua New Guinea. The negative Tajima’s *D* and Fu’s *F_S_* values showed the recent demographic expansion of *Maruca* population. The haplotype network and Automatic Barcode Gap Discovery analyses confirmed the results of phylogenetic analysis. Thus, this study confirmed the presence of three putative *Maruca* species, including one in Latin America, one in Oceania (including Indonesia) and *M*. *vitrata* in Asia, Africa and Oceania. Hence, the genetic differences in *Maruca* population should be carefully considered while designing the pest management strategies in different regions.

## Introduction

Legume pod borer, *Maruca vitrata* (F.) (syn. *M*. *testulalis*) (Lepidoptera: Crambidae), is considered as the most serious pest of food legumes in tropical Asia, sub-Saharan Africa, South America, North America, Australia and the Pacific [1]. *M*. *vitrata* can feed on at least 45 different host plant species, mostly on legumes as well as two non-legume species, in tropical Asia and sub-Saharan Africa [1–4]. It is reported as a major pest on different cultivated legume species (*Vigna unguiculata* subsp. *sesquipedalis*, *V*. *radiata*, *V*. *mungo*, *Cajanus cajan*, *Lablab purpureus*, *Phaseolus angularis*, *P*. *vulgaris*, *Sesbania cannabina*, *S*. *grandiflora* and *Glycine max*) year-round in South and Southeast Asia. There are relatively few cultivated legumes that serve as host plants for *M*. *vitrata* in sub-Saharan Africa, although cowpea (*V*. *unguiculata*) is the predominant host. The majority of *M*. *vitrata* population occur on perennial leguminous shrub or tree hosts, particularly during the main dry season [3,5]. Moths and larvae of *M*. *vitrata* are nocturnal. Female moths prefer to lay eggs on the floral buds. Larvae create webs on floral buds, flowers and pods, inside of which they feed on the various plant parts [1]. Infestation that starts in the terminal shoots but later spreads to the reproductive structures [6], is highest on flowers, followed by floral buds, pods, and leaves [1,7]. The mature larvae, especially from the third instar, are capable of damaging pods and occasionally the peduncle and stems [8]. Up to 80% of yield losses have been reported in various vegetable and grain legumes due to *M*. *vitrata* damage [9–12].

*M*. *vitrata* is believed to be the only *Maruca* species causing economic damage on food legumes. Seven other *Maruca* species *viz*., *M*. *amboinalis* (Felder 1875), *M*. *aquitilis* (Guérin-Méneville 1832), *M*. *bifenestralis* (Mabille 1880), *M*. *fuscalis* (Yamanaka 1998), *M*. *nigroapicalis* (Joannis 1930), *M*. *simialis* (Snellen 1882) and *M*. *testulalis* (Geyer, 1832) are mentioned in the genus *Maruca* [13]. However, *M*. *testulalis* was found to be synonymous with *M*. *vitrata* (Fabricius 1787). Besides *M*. *vitrata*, only two other species, *M*. *amboinalis* and *M*. *nigroapicalis*, have been described, and the latter was never found again after the first description [14–16]. They were observed in the Indo-Malaysian and Tonkin area, the most probable center of origin for the genus *Maruca*. The question that remains unanswered is whether the species *M*. *vitrata* is composed of any cryptic species. A recent study has shown evidence for the presence of multiple unique *Maruca* species or subspecies worldwide [17]. Two forms of *M*. *vitrata* were reported in Australia [18]. Whether *M*. *vitrata* is a species or species complex, has implications for the management of this pest, especially for using pheromones for monitoring and/or mass-trapping, since pheromones are an important component in integrated pest management (IPM) strategies.

(E,E)-10,12-hexadecadienal was identified as the major component of *M*. *vitrata* sex pheromone [19], whereas (E,E)-10,12-hexadecadienol, and (E)-10-hexadecenal were identified as minor components [20]. Re-examination of *M*. *vitrata* sex pheromone revealed two new components such as (*E*)-10-hexadecenol and (*Z*,*Z*,*Z*,*Z*,*Z*)-3,6,9,12,15-tricosapentaene [21]. Variations have been observed in the responses of *M*. *vitrata* males to synthetic sex pheromone lures over various geographical locations in sub-Saharan Africa and tropical Asia. A synthetic sex pheromone developed for *M*. *vitrata* consisting of (E,E)-10,12-hexadecadienal, (E,E)-10,12-hexadecadienol, and (E)-10-hexadecenal attracted male *M*. *vitrata* moths in Benin and Ghana, while (E,E)-10,12-hexadecadienal alone was most effective in Burkina Faso [22]. Neither pheromone was effective in Taiwan [23], while a variant blend was effective in India [21]. It is imperative to assess the homogeneity of *M*. *vitrata* population to develop pheromone-based control methods.

DNA polymorphism in mitochondrial and nuclear genes has been used for insect molecular systematics and diagnostics. Sequences of the mitochondrial cytochrome oxidase I and II (*cox1 and cox2*) genes and internal transcribed spacer 1 (ITS1) and 2 (ITS2) regions are commonly used to differentiate species or cryptic species within a species complex. Molecular phylogeny using these DNA sequences has been well established in thrips [24], whiteflies [25] and *M*. *vitrata* [17]. Indo-Malaysian region is considered to be the center of origin for *M*. *vitrata* [15] and there are no earlier reports available on *M*. *vitrata* in this region. Hence, the aim of our study was to assess the genetic diversity and host plant races of *M*. *vitrata* population in South and Southeast Asia and sub-Saharan Africa on different host plant species, and to determine if subspecies have been formed.

## Materials and Methods

### Ethics Statement

No specific permits were required for the described studies, and no specific permissions were required for these locations/activities. We confirm that samples were taken from non-endangered, non-protected species on open, public lands.

### Insect sampling

For this study, a total of 77 different *M*. *vitrata* population containing 686 individuals from 22 host plants covering 13 genera (*Sesbania cannabina*, *S*. *grandiflora*, *S*. *rostrata*, *S*. *vesicaria*, *Vigna unguiculata*, *V*. *unguiculata*. subsp. *sesquipedalis*, *V*. *radiata*, *V*. *angularis*, *V*. *cylindrica*, *V*. *sinensis*, *Phaseolus* sp., *P*. *vulgaris*, *Cajanus cajan*, *Psophocarpus tetragonolobus*, *Pterocarpus santalinoides*, *Canavalia* sp., *Pueraria phaseoloides*, *Dolichos lablab*, *Lonchocarpus cyanescens*, *Tephrosia bracteolata*, *Crotalaria juncea* and *Centrosema pubescens*) in 10 countries, *viz*., Bangladesh (24°12’N 89°08’E), Benin (6°45’N 2°38’E to 6°58’N 1°39’E, 7°01’N 2°37’E to 7°53’N 2°05’E, 8°05’N 2°32’E to 8°51’N 2°36’E, 9°17’N 2°52’E to 9°44’N 1°40’E, 58’68N 3°15’16E and 11°37’81N 3°10’74E), Indonesia (7°42’S 110°31’E), India (11°61’N 78°01’E, 12°95’N 77°63’E, 23°33’N 85°31’E, 22°55’N 72°95’E and 30°91’N 75°85’E), Kenya (0°19’S 34°11’E to 0°80’S 34°72’E and 0°42’S 37°57’E to 0°57’S 37°62’E), Lao PDR (16°30’N 104°49’E to 16°58’N 104°44’E, 17°03’N 104°46’E to 17°51’N 102°38’E, 18°21’N 102°32’E to 18°25’N 102°28’E and 19°24’N 102°06’E to 19°56’N 102°13’E), Malaysia (2°33’N 102°21’E, 2°78’N 101°99’E, 6°19’N 102°11’E, 3°00’N 101°71’E, 3°06’N 101°61’E, 3°42’N 102°50’E, 3°49’N 101°94’E, 4°47’N 101°47’E and 5°19’N 116°15’E to 5°89’N 116°78’E), Taiwan (22°46N 120°29’E to 23°12N 120°30’E), Thailand (13°53’N 100°01E, 14°01N 99°57’E, 14°10N 100°46’E and 15°12N 99°51’E) and Vietnam (14°50’N 105°49’E, 20°06’N 105°05’E to 21°23’N 106°00”E, 11°16’N 108°05”E and 10°42’N 105º26”E) were collected from the field, and preserved in 95% ethanol.

### DNA Extraction

We followed two methods using locally available kits following manufacturers’ instructions with slight modifications. In the first method, DNA was extracted from a single insect using Easy DNA High-speed Extraction Tissue Kit (Saturn Biotech, Taiwan). The whole insect was placed on Whatman filter paper, washed with double distilled water, and allowed to dry for 5 min. The head was dissected using a sterile knife and forceps, and transferred to eppendorf tubes containing 32 μl of Solution 1A and 8 μl of Solution 1B. The tubes were vortexed for 10 s and moved to the dry heater for 20 min at 95 °C. After cooling, 10 μl of Solution 2 was added, and the mixture was stored in aliquots (20 μl and 30 μl) at -20 °C. In the second method, DNA was extracted from a single insect using BuccalAmp DNA Extraction Kit (Epicentre, through Bio-Genesis Technologies, Inc., Taipei, Taiwan). In this method, a 0.5–1 cm long piece was cut below the head and transferred to eppendorf tubes containing 50 μl extraction solution, each. The samples were vortexed for 15 s, incubated in a water bath for 30 min at 65 °C, vortexed again for 15 s and moved to a dry heater for 15 min at 98 °C. The DNA solution was treated with RNase and stored in aliquots (20 μl and 30 μl) at -20 °C.

### Polymerase chain reaction (PCR) and DNA sequencing

The universal *cox1* primer pair (HC02198 5Rev'-TAA ACT TCA GGG TGA CCA AAA AAT CA-3' and LCO1490: 5'For-GGT CAA CAA ATC ATA AAG ATA TTG G-3') reported by Folmer et al. [26] was used to amplify the partial sequence of the *cox1* gene of single *M*. *vitrata* larvae. DNA was quantified using spectrophotometry and analytical gel densitometry before performing PCR. PCR was performed in a total reaction volume of 25 μl containing 75 ng of DNA template, 1X PCR buffer, 0.6 mM MgCl_2_, 0.15 mM dNTPs, 0.5 μM of each forward and reverse primer, 0.015 unit/μl *Taq* polymerase (Super-Therm Gold DNA Polymerase, Catalogue No. JMR851, JMR Holdings, UK) and 12.75 μl of sterile distilled water. PCR was performed in a MJ Research Thermocycler (PTC200 DNA Engine Cycler, Bio-Rad Laboratories, Inc.) following the profile: 95 °C for 10 min followed by 4 cycles of 95 °C for 30 s, 55 °C for 45 s and 72 °C for 1.30 min, followed by 30 cycles of 95 °C for 30 s, 50 °C for 45 s, 72 °C for 45 s with the final extension at 72 °C for 8 min. After amplification, 5 μl of the PCR product of each sample was analyzed by electrophoresis on 1.5% agarose gels containing ethidium bromide. Bands were revealed and photographed under ultraviolet light. After electrophoresis, the remaining PCR products were used for sequencing with the previously mentioned forward and reverse primers, using **ABI 3730XL systems** at Genomics Bioscience and Technology Company Limited, Taiwan.

### Molecular divergence and population genetic analyses

The *cox1* sequences were aligned and edited using BioEdit version 7.0 [27]. The obtained sequences were aligned with the mitochondrion genome reference sequences from National Center for Biotechnology Information (NCBI) GenBank (GenBank accession numbers HM751150, KJ466365 and NC024099) to confirm that the amplified gene region is located in the mitochondria only. Subsequently, the sequences were examined for polymorphism among the *Maruca* population collected from different locations or host plants. Reference sequences from *M*. *vitrata* population from Latin America [28] and Oceania [29] were obtained from the NCBI GenBank database and the International Barcode of Life project (iBOL). The number of variable nucleotide sites, number of haplotypes, nucleotide diversity and haplotype diversity were calculated for investigating the *cox1* sequence diversity using DnaSP 5.10 software [30]. Statistical tests of Tajima’s *D* and Fu’s *F*_*S*_ values were used to detect the deviation from the neutral model of evolution using DnaSP 5.10. Tajima’s *D* uses mutation frequencies in the sequences to identify if a population has undergone a recent population expansion event, and is determined by the difference between average number of nucleotide differences and the number of segregating sites estimated from pair-wise comparisons [31]. Fu’s *F*_*S*_ test uses information from the haplotype distribution in a sample. The test estimates the probability of observing a random sample with equal or less singletons than the observed given a level of diversity. The test is based on the infinite site mutation model, and assumes that all of the alleles are selectively neutral.

The genetic structure of *M*. *vitrata* population based on *cox1* sequences was examined by Analysis of Molecular Variance (AMOVA) using Arlequin 2.001 [32,33]. This method was used to partition the genetic variance within population, among population within groups and among groups. The population was grouped either by geographical location (regions) based on phylogenetic tree or by host plant species (Table 1). Due to the strong differentiation found among the reference population of *Maruca* in Oceania and Latin America, we conducted another AMOVA on the species groups based on Automatic Barcoding Gap Discovery (ABGD) tree. Levels of significance were determined through 1000 random permutation replicates. Pair-wise *F*_*ST*_ values used to appraise the genetic structure among population were obtained with 1000 permutations and at the significance level of 0.05 using the Kimura 2-parameter (K2P) model [34]. A Mantel test was performed with the web tool isolation by distance (version IBD 3.23) [35] to examine the correlation between genetic differentiation (*F*_*ST*_) and geographic distance. The Euclidean distances between populations were measured based on a strategic central location in a collection region in the country. For the continent / region based analysis, we chose a strategic central country in a continent / region to measure the shortest straight distance.

**Table 1:** List of identified *Maruca vitrata* haplotypes with their geographical origin and host plants.

Haplotype	Representative sample	Haplotype frequency	NCBI GenBank (accession number)	Population
1	DL-9	1	KM987699	Lao PDR (*Sesbania vesicaria*)
2	HL-1	2	KM987700	Lao PDR (*Phaseolus* sp.)
3	CD-8	1	KM987701	India (*Vigna unguiculata*)
4	BV-2	1	KM987702	Vietnam (*V*. *cylindrica*)
5	VL-3	1	KM987703	Lao PDR (*V*. *unguiculata* subsp. *sesquipedalis*)
6	VLX-3	2	KM987704	Lao PDR (*V*. *unguiculata* subsp. *sesquipedalis*)
7	VV5-5	1	KM987705	Vietnam (*V*. *unguiculata* subsp. *sesquipedalis*)
8	SW-1	225	KM987706	Bangladesh (*V*. *unguiculata*), Benin (*Pterocarpus santalinoides*), India (*V*. *unguiculata*; *Phaseolus vulgaris*; *Cajanus cajan*; *Dolichos lablab*), Indonesia (*Sesbania grandiflora*), Lao PDR (*S*. *vesicaria*; *V*. *radiata*; *C*. *cajan*; *V*. *unguiculata* subsp. *sesquipedalis*), Malaysia (*V*. *sinensis*; *P*. *vulgaris*; *V*. *unguiculata* subsp. *sesquipedalis*), Taiwan (*Sesbania cannabina*; *S*. *grandiflora*; *Canavalia* sp.; *V*. *unguiculata*; *V*. *unguiculata* subsp. *sesquipedalis; C*. *cajan*; *D*. *lablab*; *V*. *angularis*), Thailand (*S*. *grandiflora*; *V*. *radiata*; *V*. *unguiculata* subsp. *sesquipedalis*; *Psophocarpus tetragonolobus*), Vietnam (*V*. *cylindrica*; *S*. *grandiflora*; *V*. *radiata*; *P*. *vulgaris*; *V*. *unguiculata* subsp. *sesquipedalis*; *P*. *tetragonolobus*)
9	MVM-1	2	KM987707	Vietnam (*V*. *Radiata*), Lao PDR (*V*. *unguiculata* subsp. *sesquipedalis*)
10	YW-6	1	KM987708	Taiwan (*D*. *lablab*)
11	VV14-2	1	KM987709	Vietnam (*V*. *unguiculata* subsp. *sesquipedalis*)
12	AW-5	1	KM987710	Taiwan (*Canavalia* sp.)
13	VLH-2	1	KM987711	Lao PDR (*V*. *unguiculata subsp*. *sesquipedalis*)
14	BV-1	1	KM987712	Vietnam (*V*. *cylindrica*)
15	CG-7	1	KM987713	Bangladesh (*V*. *unguiculata*)
16	VLV-2	1	KM987714	Lao PDR (*V*. *unguiculata* subsp. *sesquipedalis*)
17	MVM-5	1	KM987715	Vietnam (*V*. *radiate*)
18	IM-5	3	KM987716	Malaysia (*V*. *sinensis*)
19	OVB-2	1	KM987717	Vietnam (*P*. *vulgaris*)
20	BV-12	1	KM987718	Vietnam (*V*. *cylindrica*)
21	HL-7	2	KM987719	Lao PDR (*Phaseolus* sp., *V*. *unguiculata* subsp. *sesquipedalis*)
22	HL-8	2	KM987720	Lao PDR (*Phaseolus* sp.), Taiwan (*V*. *angularis*)
23	IMS-9	32	KM987721	Benin (*Canavalia* sp., *V*. *unguiculata*, *Pueraria phaseoloides*, *Sesbania rostrata*, *Tephrosia bracteola*, *Pterocarpus santalinoides*), Kenya (*V*. *unguiculata*, *P*. *phaseoloides*, *Crotalaria juncea*, *P*. *vulgaris*, *D*. *lablab*), Lao PDR (*V*. *unguiculata* subsp. *sesquipedalis*), Malaysia (*V*. *sinensis*), Taiwan (*V*. *unguiculata*, *V*. *unguiculata* subsp. *sesquipedalis*)
24	TB-7	1	KM987722	Benin (*T*. *bracteola*)
25	PKM-5	1	KM987723	Kenya (*C*. *cajan*)
26	TB-1	1	KM987724	Benin (*T*. *bracteola*)
27	LB-10	1	KM987725	Benin (*Lonchocarpus cyanesens*)
28	AB-1	1	KM987726	Benin (*Canavalia* sp.)
29	EB-5	1	KM987727	Benin (*P*. *phaseoloides*)
30	RB-10	15	KM987728	Benin (*Canavalia* sp., *V*. *unguiculata*, *P*. *phaseoloides*, *L*. *cyanesens*, *S*. *rostrata*, *T*. *bracteola*, *P*. *santalinoides*), Kenya (*V*. *unguiculata*, *C*. *cajan*, *P*. *vulgaris*)
31	UB-9	2	KM987729	Benin (*S*. *rostrata*, *P*. *santalinoides*)
32	TB-6	1	KM987730	Benin (*T*. *bracteola*)
33	CB-9	2	KM987731	Benin (*V*. *unguiculata*, *L*. *cyanesens*)
34	NK-8	4	KM987732	Kenya (*C*. *cajan*, *P*. *vulgaris*, *Centrosema pubescens*, *T*. *bracteola*)
35	YK-4	143	KM987733	Benin (*Canavalia* sp., *V*. *unguiculata*, *P*. *phaseoloides*, *L*. *cyanesens*, *S*. *rostrata*, *T*. *bracteola*, *P*. *santalinoides*), Kenya (*V*. *unguiculata*, *P*. *phaseoloides*, *C*. *juncea*, *C*. *pubescens*, *P*. *vulgaris*, *C*. *cajan*, *T*. *bracteola*, *D*. *lablab*)
36	VL-11	1	KM987734	Lao PDR (*V*. *unguiculata* subsp. *sesquipedalis*)
37	IMU-9	2	KM987735	Malaysia (*V*. *sinensis*)
38	OV-5	2	KM987736	Vietnam (*P*. *vulgaris*, *V*. *cylindrica*)
39	ODB-6	29	KM987737	India (*V*. *unguiculata*, *P*. *vulgaris*), Lao PDR (*Phaseolus* sp., *V*. *unguiculata* subsp. *sesquipedalis*), Malaysia (*V*. *sinensis*), Taiwan (*S*. *grandiflora*, *V*. *radiata*, *D*. *lablab*), Thailand (*S*. *grandiflora*, *V*. *unguiculata* subsp. *sesquipedalis*, *P*. *tetragonolobus*), Vietnam (*V*. *radiata*; *V*. *unguiculata* subsp. *sesquipedalis*)
40	YDR-1	6	KM987738	India (*D*. *lablab*)
41	WT-7	1	KM987739	Thailand (*P*. *tetragonolobus*)
42	VLS-7	1	KM987740	Lao PDR (*V*. *unguiculata* subsp. *sesquipedalis*)
43	VW-13	1	KM987741	Taiwan (*V*. *unguiculata* subsp. *sesquipedalis*)
44	GV-3	1	KM987742	Vietnam (*S*. *grandiflora*)
45	XM-6	1	KM987743	Malaysia (*V*. *unguiculata* subsp. *sesquipedalis*)
46	VT-1	20	KM987744	India (*D*. *lablab*), Lao PDR (*V*. *unguiculata* subsp. *sesquipedalis*), Taiwan (*S*. *grandiflora*, *V*. *radiata*, *V*. *unguiculata*), Thailand (*S*. *grandiflora*, *V*. *radiata*, *V*. *unguiculata* subsp. *sesquipedalis*), Vietnam (*V*. *radiata*, *P*. *vulgaris*, *V*. *unguiculata* subsp. *sesquipedalis*)
47	YDR-6	1	KM987745	India (*D*. *lablab*)
48	VLH-9	2	KM987746	Lao PDR (*V*. *unguiculata* subsp. *sesquipedalis*), Malaysia (*V*. *sinensis*)
49	CN-5	2	KM987747	Indonesia (*V*. *unguiculata*)
50	XM-8	1	KM987748	Malaysia (*V*. *unguiculata* subsp. *sesquipedalis*)
51	VVB-2	1	KM987749	Vietnam (*V*. *unguiculata* subsp. *sesquipedalis*)
52	MT-3	1	KM987750	Thailand (*V*. *radiate*)
53	VLS-9	1	KM987751	Lao PDR (*V*. *unguiculata* subsp. *sesquipedalis*)
54	PM-1	143	KM987752	Bangladesh (*V*. *unguiculata*), India (*V*. *unguiculata*; *C*. *cajan*), Indonesia (*S*. *grandiflora*, *V*. *unguiculata*), Lao PDR (*S*. *vesicaria*, *Phaseolus* sp., *V*. *radiata*, *V*. *unguiculata* subsp. *sesquipedalis*), Malaysia (*V*. *sinensis*, *P*. *vulgaris*, *C*. *cajan*, *V*. *unguiculata* subsp. *sesquipedalis*), Taiwan (*S*. *cannabina*, *Canavalia* sp., *V*. *radiata*, *V*. *unguiculata*, *V*. *unguiculata* subsp. *sesquipedalis*, *D*. *lablab*), Thailand (*V*. *radiata*; *P*. *tetragonolobus*), Vietnam (*V*. *cylindrica*, *V*. *radiata*, *P*. *vulgaris*, *V*. *unguiculata* subsp. *sesquipedalis*, *P*. *tetragonolobus*)
55	OM-2	1	KM987753	Malaysia (*P*. *vulgaris*)
56	MVB-4	1	KM987754	Vietnam (*V*. *radiate*)
57	MV-11	1	KM987755	Vietnam (*V*. *radiate*)
58	HL-6	2	KM987756	Lao PDR (*Phaseolus* sp.), Taiwan (*V*. *unguiculata* subsp. *sesquipedalis*)
59	ZW-2	1	KM987757	Taiwan (*V*. *angularis*)
60	ML-4	1	KM987758	Lao PDR (*V*. *radiata*)
61	PW-4	1	KM987759	Taiwan (*C*. *cajan*)
62	WT-3	1	KM987760	Thailand (*P*. *tetragonolobus*)
63	VLN-7	1	KM987761	Lao PDR (*V*. *unguiculata* subsp. *sesquipedalis*)
64	GW-10	1	KM987762	Taiwan (*S*. *grandiflora*)

### Phylogenetic, species delineation and haplotype network analyses

The FASTA formatted *cox1* sequences were imported into the MEGA 5 software package sequence alignment application and a multiple sequence alignment was performed with ClustalW algorithm using default parameters [36]. The insects that showed 100% nucleotide similarities were designated as a single *cox1* haplotype and the others showing different sequence polymorphisms were designated as different *cox1* haplotypes (Table 1). *cox1* sequences of *M*. *vitrata* population from Australia, China, Indonesia, Pakistan, Philippines, Papua New Guinea, sub-Saharan Africa (Cameroon, Gabon, Ghana, Kenya, Nigeria), and Latin America (Argentina, Bolivia, Brazil, Costa Rica, French Guiana, Mexico, Panama) obtained from i-BOL as well as from NCBI GenBank were used as reference sequences. The aligned sequences were used for phylogenetic analysis.

Maximum likelihood (ML) and Maximum parsimony (MP) phylogenetic analyses were used to identify major clades and to evaluate the relationships among the haplotypes of the *cox1* sequences. The appropriate model of sequence evolution, including model parameters, was calculated using corrected Akaike Information Criterion (AICc value) with MEGA 5, and resulted in T92+G (Tamura 3 with Gamma shape parameter) as the best model [36]. The model was also selected based on partitioning by codon position. With those settings, a heuristic search was performed (nearest neighbor interchange algorithm starting tree obtained via neighbor joining). For ML analysis, non-uniformity of evolutionary rates among sites was modeled by using a discrete Gamma distribution (+G) with 5 rate categories. Whenever applicable, estimates of gamma shape parameter were included. The clustering probabilities of each resulting phylogenetic tree node were statistically tested by a bootstrap method consisting of 1000 replicates. MP analyses were performed in MEGA 5 employing a heuristic search, using tree bi-section-reconnection (TBR) branch swapping, with simple, closest, random (50 replications) taxon addition. The initial unweighted parsimony analysis of the full nucleotide dataset also included 1000 bootstrap replications. Posterior probabilities were calculated by constructing a 50% majority rule consensus tree of the stationary trees. All trees were rooted by the outgroup *Ostrinia nubilalis*.

To validate the results of phylogenetic analysis, we evaluated the primary species hypothesis using a molecular species delineation method, Automatic Barcode Gap Discovery (ABGD). ABGD is an automated procedure that clusters sequences into candidate species based on pairwise distances by detecting differences between intra- and interspecific variation without *a priori* species hypothesis [37]. The program requires two user-specified values: P (prior limit to intraspecific diversity) and X (proxy for minimum gap width). We analyzed the *coxI* sequences in the web-server of ABGD http://wwwabi.snv.jussieu.fr/public/abgd/abgdweb.html using the Kimura K80 model, a default gap width of 1.55 and the P value from 0.001 to 0.1.

We also examined the genealogical relationships among *M*. *vitrata cox1* sequences by establishing a median-joining haplotype network with the software Network 4.6 (Fluxus Technology Ltd, Suffolk, UK) using an epsilon value of 10 and maximum parsimony post-processing that removed superfluous nodes and links [38], and also downweighting the hypervariable characters. Transversions were weighted three times as high as transitions.

## Results

### *cox1* haplotype variation in *M*. *vitrata* population

The universal *cox1* primer pair (HC02198 and LCO1490) successfully amplified PCR products of 709 bp size in different *M*. *vitrata* population. Although the sequence alignment and editing resulted in a consensus sequence of 626 bp across all *M*. *vitrata* population, we used 615 bp consensus sequences to maximize the number of possible reference sequences to be included in this study. The sequence data of haplotypes identified in the current study were submitted to the NCBI GenBank (accession number: KM987699–KM987762). Analysis of the average nucleotide compositions in the *M*. *vitrata cox1* gene fragments showed high A+T content (70.22%) and low content of G+C (29.78%), which was consistent with the AT- rich nature of the *cox1* gene. A total of 50 substitution mutations were detected, with 45 being transition mutations (ratio of transitions to transversions = ts/tv = *k* = 3.17), resulting in a transition to transversion bias of (*R*) = 2.716.

In total, 64 *cox1* haplotypes were identified in 686 *M*. *vitrata* individuals based on sequence similarity at 47 polymorphic sites, of which 17 were parsimony informative. The largest haplotype (Haplotype 8) contain 225 *M*. *vitrata* individuals, of which all samples were collected from Asian countries, except one from Benin (Table 1). Although Haplotype 8 had more *M*. *vitrata* individuals, most of them were sampled from Asian countries, it had only two out of 20 individuals originating from Magelang, central Java, Indonesia. Two haplotypes (Haplotype 35 and Haplotype 54), each with 143 *M*. *vitrata* individuals, formed the second largest haplotype group. Haplotype 35 contains *M*. *vitrata* from sub-Saharan Africa, whereas Haplotype 54 had *M*. *vitrata* from Asian countries. Interestingly, 16 out of 20 *M*. *vitrata* individuals collected from central Java in Indonesia fell only within Haplotype 54. The third largest haplotype (Haplotype 23) containing 32 *M*. *vitrata* individuals, mostly individuals from Africa, also included a few *M*. *vitrata* individuals from Lao PDR, Malaysia and Taiwan. The mitochondrion genome reference sequence from NCBI GenBank (HM751150) also aligned with Haplotype 23. Haplotype 39 and Haplotype 46 contained 29 and 20 individuals, respectively, all of which had been collected only from target Asian countries, except Indonesia (Central Java). Haplotype 30 contained 15 *M*. *vitrata* individuals that originated from African countries. Six out of 10 *M*. *vitrata* individuals originating from Jharkhand, India formed a unique haplotype (Haplotype 40). Four *M*. *vitrata* individuals from Kenya and three from Malaysia formed haplotypes 34 and 18, respectively. A total of 12 haplotypes shared two individuals, and the remaining 42 haplotypes were unique, with only single insects.

Nucleotide diversity is used to measure the degree of polymorphism within a population [39]. The nucleotide diversity of *M*. *vitrata* population from Kenya was the lowest (0.00087) with Vietnam being the highest (0.00214), followed by Malaysia, Thailand, Benin and Lao PDR (Table 2). The total nucleotide diversity of all *M*. *vitrata* population from sampled countries was 0.00309. The haplotype diversity is a measure of the uniqueness of a particular haplotype in a given population [40]. The haplotype diversity value was lowest in Kenya, followed by Indonesia; it was highest in Thailand, followed by Benin and Vietnam. The total haplotype diversity value of all *M*. *vitrata* population from sampled countries was 0.801 (Table 2). The lowest haplotype diversity and nucleotide diversity both occurred in the Kenya population. In contrast, although the nucleotide diversity was highest in Vietnam, the highest haplotype diversity occurred in Thailand. In a total of 64 haplotypes, only 8% of the haplotypes were shared among multiple countries. Two haplotypes (30 and 35) were shared only by Kenya and Benin and are specific for the African continent. Similarly, Vietnam and Lao PDR, as well as Lao PDR and Malaysia, shared one haplotype each. The remaining haplotypes were specific to a particular country. The number of haplotypes was highest in Lao PDR, followed by Vietnam, and lowest in Indonesia (Central Java). Since very few samples were collected from Bangladesh, they were combined with samples from India.

**Table 2:** List of number of samples studied, number of haplotypes, haplotype diversity (*h*), nucleotide diversity (π), Tajima’s *D* and Fu’s *F*_*S*_ tests for *Maruca vitrata* populations from ten countries in South and Southeast Asia, and sub-Saharan Africa.

Country	No. of samples	No. of haplotypes	Haplotype diversity (*h*)	Nucleotide diversity (π)	Tajima’s *D*	Tajima's D (NonSyn/Syn) ratio	Fu’s *F*_*S*_
India (including Bangladesh)	57	8	0.619	0.00180	-0.66314	2.74183	-2.319
Thailand	41	7	0.712	0.00189	-0.41528	-	-1.672
Lao PDR	98	21	0.645	0.00185	-1.90868*	-	-19.581**
Vietnam	107	17	0.700	0.00214	-1.55724	0.98528	-10.426**
Malaysia	81	10	0.613	0.00194	-0.85345	-	-3.364*
Indonesia (Central Java)	20	3	0.358	0.00091	-0.87550	0.76967	0.031
Taiwan	83	13	0.641	0.00164	-1.28504	0.81737	-8.044**
Benin	69	12	0.703	0.00188	-0.98528	-1.13421	-6.165**
Kenya	130	5	0.289	0.00087	-0.49067	-0.36717	-1.045
All countries	686	64	0.801	0.00309	-1.96635*	0.66033	-74.078**

** values were significant at P < 0.01

*** values were significant at P < 0.001

When the *M*. *vitrata* samples were analyzed by continent, the highest haplotype numbers were recorded in Asia, and the lowest haplotype numbers occurred in Oceania (Table 3). However, Africa had fewer haplotypes than Latin America, although Africa had more than twice the number of *M*. *vitrata* samples as Latin America. Both Africa and Latin America had less haplotype diversity than Oceania and Asia. The nucleotide diversity was also less for both Africa and Latin America, whereas it was almost 20- to 30-fold higher for Oceania.

**Table 3:** List of number of samples studied, number of haplotypes, haplotype diversity (*h*), nucleotide diversity (π), Tajima’s *D* and Fu’s *F*_*S*_ tests for *Maruca vitrata* populations from four selected continents / regions.

Continent / Region	No. of samples	No. of haplotypes	Haplotype diversity (*h*)	Nucleotide diversity (π)	Tajima’s *D*	Tajima's D (NonSyn/Syn) ratio	Fu’s *F*_*S*_
Africa	199	14	0.464	0.00133	-1.21271	-0.71451	-9.043
Asia	490	54	0.701	0.00311	-2.45557***	0.66712	-57.941
Oceania	37	10	0.806	0.03816	2.66440**	-0.07254	13.618
Latin America	93	16	0.384	0.00193	-2.59704***	0.60453	-10.779
All continents / regions	819	81	0.802	0.01223	-1.53255	1.17793	-30.284

** values were significant at P < 0.01

*** values were significant at P < 0.001

Tajima’s *D* test did not show any positive value and the values were not significant, except for Lao PDR population or the overall population (Table 2). When the samples were analyzed by continent, Tajima’s *D* test showed negative values for all except the Oceania samples which have significant positive values, whereas the values were highly significant for both Asia and Latin America (Table 3). The negative Tajima’s *D* values indicated that the *M*. *vitrata* population in the target countries or in Asia, Africa and Latin America began to expand recently, and they provide evidence for purifying selection at this locus. However, significantly positive Tajima’s *D* value for Oceania indicated that the *M*. *vitrata* population may have suffered a recent sharp decline in its size (bottleneck).

Similarly, apart from the Indonesia (Central Java) population (Table 2) or the Oceania population (Table 3), all other population showed negative values for Fu’s *F*_*S*_ test with or without significance. Thus, besides Tajima’s *D*, a negative value of Fu’s *F*_*S*_ for most of our studied population is evidence for a possible recent population expansion or genetic drift due to random sampling. A positive value of Fu’s *F*_*S*_ for Indonesia (Central Java) or Oceania population is evidence for the deficiency of alleles due to a recent population decrease.

A 208 residue *cox1* amino acid (aa) sequence was derived from *M*. *vitrata* sequence data, from which multiple sequence alignments predicted four non-synonymous aa changes, with the serine to glycine mutation at aa position 109, present only in three *M*. *vitrata* individuals (one from Vietnam and two from Indonesia (Central Java)). A change from isoleucine to valine at aa position 117 was observed in an individual from India. Another change from leucine to methionine at aa position 146 was mainly observed in African population in 57% of Benin samples and 87% of Kenya samples, besides two Asian individuals (each one from Lao PDR and Taiwan). The fourth mutation at aa position 155 with the isoleucine to valine was observed in a Vietnam individual. Except for these four non-synonymous mutations, all the other mutations were predicted to be silent.

### F-statistics (Fst) and Analysis of Molecular Variance (AMOVA)

The *F*_*ST*_ values of all population pairwise comparisons were ranged from -0.001 to 0.85 (Table 4). Negative *F*_*ST*_ values can be interpreted as no genetic differences between the two populations compared, due to imprecision of the algorithm used [41]. Based on the negative *F*_*ST*_ values, India (South), Thailand and Lao PDR population are not significantly different from the India (Gujarat, GJ) population. Lao PDR (Savannakhet, SK) population is not significantly different from the India (South) population. Similarly, Bangladesh, Lao PDR (Vientiane, VN), Vietnam and Taiwan population are not significantly different from the India (Punjab, PB) population, and Thailand, Lao PDR (VN), Vietnam and Taiwan population are not significantly different from the Bangladesh population. Lao PDR (VN) population is not significantly different from Taiwan population. Within-country population from Benin, Kenya, Lao PDR, Malaysia and Vietnam are homogeneous and not significantly different from each other. The genetic difference of both the Benin and Kenya population from all the Asian population was highly significant based on pairwise F_*ST*_ values (0.44–0.85; p<0.01). It is interesting to note that the genetic difference of Benin (South and Central) population from the Kenyan population was highly significant based on pairwise F_*ST*_ values (0.09–0.26; p<0.01). However, Benin (North) population is not significantly different from Kenya (Eastern) whereas it is significantly different from Kenya (Nyanza, NYZ) (0.07; p<0.05). Among the Asian population, the genetic difference of the India (Jharkhand, JK) population from all other population was highly significant. Similarly, the genetic difference of the Indonesia (Central Java, JV) population from all other population, except the Malaysia (East) population, was also highly significant (0.11–0.74).

**Table 4:** Pairwise *FS*_*T*_ values (below diagonal) and distance matrix (above diagonal; in the unit of kilometer) comparing populations of *Maruca vitrata* (country based analysis).

Population	1	2	3	4	5	6	7	8	9	10	11	12	13	14	15	16	17	18	19	20
1. India GJ	0.0000	1,334	972	1,272	1,670	3,001	3,395	3,134	3,396	3,948	3,691	5,079	3,740	5,260	4,823	7,723	7,443	7,693	4,917	4,627
2. India South	-0.04	0.0000	2,156	1,508	1,807	2,379	2,920	2,721	3,130	3,270	2,962	4,306	2,731	4,139	4,620	8,319	8,127	8,344	5,042	4,699
3. India PB	0.11	0.26**	0.0000	1,260	1,528	3,097	3,336	3,059	3,184	3,991	3,796	5,130	4,070	5,628	4,455	8,034	7,691	7,961	5,612	5,356
4. India JK	0.26**	0.40**	0.40**	0.0000	558	1,855	2,166	1,895	2,127	2,777	2,559	3,920	2,814	4,372	3,555	8,988	8,697	8,951	6,126	5,818
5. Bangladesh	0.01	0.13	-0.09	0.34**	0.0000	1,580	1,818	1,542	1,737	2,463	2,273	3,602	2,636	4,181	3,151	9,379	9,080	9,337	6,528	6,218
6. Thailand	-0.02	0.04	0.03	0.30**	-0.02	0.0000	587	523	999	948	704	2,081	1,184	2,650	2,352	10,638	10,401	10,637	7,417	7,071
7. Laos SK	-0.04	-0.01	0.09*	0.33**	0.01	0.02	0.0000	277	512	711	686	1,806	1,494	2,742	1,765	11,099	10,836	11,082	7,961	7,619
8. Laos VN	-0.001	0.05	-0.004	0.34**	-0.06	0.01	-0.01	0.0000	480	971	888	2,081	1,614	2,954	1,912	10,847	10,577	10,825	7,754	7,416
9. Vietnam North	0.11*	0.18**	-0.06	0.38**	-0.03	0.05*	0.12**	0.05*	0.0000	1,136	1,185	2,103	2,006	3,210	1,493	11,115	10,817	11,074	8,133	7,802
10. Vietnam Binh Thuan	0.15**	0.25**	-0.06	0.41**	-0.02	0.08*	0.16**	0.07*	-0.02	0.0000	324	1,143	1,113	2,078	1,843	11,560	11,344	11,576	8,273	7,920
11. Vietnam An Giang	0.10*	0.19**	-0.06	0.38**	-0.05	0.03	0.11**	0.03	-0.02	-0.02	0.0000	1,389	871	2,066	2,117	11,272	11,056	11,284	7,953	7,600
12. Malaysia East	0.45**	0.60**	0.11	0.57**	0.22	0.30**	0.40**	0.27**	0.09	0.05	0.12	0.0000	1,708	1,612	1,990	12,623	12,428	12,649	9,202	8,843
13. Malaysia West	0.29**	0.36**	0.02	0.50**	0.10	0.20**	0.29**	0.18**	0.04*	0.02	0.06*	-0.03	0.0000	1,558	2,956	10,982	10,829	11,032	7,502	7,141
14. Indonesia JV	0.65**	0.74**	0.38**	0.71**	0.50**	0.48**	0.56**	0.46**	0.27**	0.25**	0.33**	0.03	0.11**	0.0000	3,562	12,085	12,017	12,183	8,468	8,107
15. Taiwan	0.02	0.07**	-0.01	0.40**	-0.05**	0.01	0.03*	-0.01	0.04*	0.06*	0.02	0.29**	0.18**	0.47**	0.0000	12,484	12,144	12,415	9,623	9,294
16. Benin South	0.60**	0.67**	0.54**	0.68**	0.55**	0.59**	0.60**	0.54**	0.52**	0.53**	0.55**	0.57**	0.52**	0.64**	0.58**	0.0000	514	286	3,640	3,995
17. Benin North	0.61**	0.72**	0.54**	0.66**	0.54**	0.59**	0.61**	0.54**	0.51**	0.52**	0.55**	0.58**	0.52**	0.68**	0.60**	-0.02	0.0000	291	3,671	4,012
18. Benin Central	0.52**	0.64**	0.44**	0.61**	0.44**	0.52**	0.53**	0.46**	0.44**	0.44**	0.47**	0.49**	0.45**	0.60**	0.52**	-0.02	-0.01	0.0000	3,778	4,127
19. Kenya NYZ	0.82**	0.84**	0.79**	0.85**	0.80**	0.77**	0.76**	0.74**	0.71**	0.74**	0.77**	0.80**	0.71**	0.82**	0.74**	0.14**	0.12*	0.26**	0.0000	363
20. Kenya Eastern	0.78**	0.82**	0.74**	0.81**	0.75**	0.73**	0.73**	0.70**	0.67**	0.69**	0.72**	0.76**	0.67**	0.79**	0.71**	0.09**	0.07	0.19**	-0.01	0.0000

* *F*_*ST*_ values were significant at *P* < 0.05

** highly significant at *P* < 0.01

When the samples were analyzed by continent, no negative *F*_*ST*_ values were found between any of the samples (Table 5). It can be interpreted that significant genetic differences exist among the population from different continents / larger geographical regions. Based on the significantly higher and positive *F*_*ST*_ values (0.9370–0.9690; p<0.01), African *M*. *vitrata* population have greater genetic differentiation from the Oceania (PNG) and Latin American population. A significantly high (p<0.01) and positive *F*_*ST*_ value of >0.5 for South Asia *M*. *vitrata* population compared to Benin, and all Asian *M*. *vitrata* population compared to Kenya *M*. *vitrata* population suggest considerable genetic differentiation from them. African *M*. *vitrata* population has little genetic differentiation from Southeast Asian and a few Australian populations.

**Table 5:** Pairwise *FS*_*T*_ values (below diagonal) and distance matrix (above diagonal; in the unit of kilometer) comparing populations of *Maruca vitrata* (continent or larger geographical region based analysis).

Populations	1	2	3	4	5	6	7	8	9
1. Asia (South)	0.0000	1,855	3,555	8,951	5,818	8,124	7,292	16,559	16,135
2. Asia (Southeast)	0.1040	0.0000	2,352	10,637	7,071	6,269	5,498	18,085	17,245
3. Asia (East)	0.0363*	0.0351**	0.0000	12,415	9,294	5,509	4,201	19,303	15,386
4. Africa (Benin)	0.5006**	0.4033**	0.4719**	0.0000	4,127	16,025	16,.049	7,608	9,501
5. Africa (Kenya)	0.7226**	0.6154**	0.7044**	0.1677**	0.0000	11,913	12,044	11,017	13,616
6. Oceania (Australia)	0.2525**	0.4524**	0.2892**	0.3094**	0.5056**	0.0000	1,611	14,964	14,468
7. Oceania (PNG)	0.9430**	0.9629**	0.9536**	0.9370**	0.9664**	0.5915**	0.0000	16,115	14,373
8. Latin America (Others)	0.9578**	0.9472**	0.9608**	0.9402**	0.9690**	0.6203**	0.8946**	0.0000	3,971
9. Latin America (Costa Rica)	0.9543**	0.9467**	0.9564**	0.9446**	0.9641**	0.7755**	0.9478**	0.0373	0.0000

* *F*_*ST*_ values were significant at *P* < 0.05

** highly significant at *P* < 0.01

AMOVA analysis was performed with the population grouped as a single group by geographical distribution (countries and continents) as well as host plants. There is relatively little differentiation among population within the same country (Φ_SC_ = 0.0310), among population within continent (Φ_SC_ = 0.3905) or among host plant population within countries (Φ_SC_ = 0.0354) (Tables 6, 7 and 8). There is substantial genetic differentiation among *M*. *vitrata* population in four selected continents / regions (Φ_CT_ = 0.7547). In fact, differences within population in various continents / regions alone is nearly responsible for all of the differences (Φ_ST_ = 0.8505). However, both the differences between population in different countries or host plants, and the differences within all population in various countries or host plants, are almost equally responsible for all of the differences when the population were analyzed on country or host plant grouping. Thus, AMOVA analysis revealed that most of the genetic variation occurred among *M*. *vitrata* population from different continents / regions (75.47%). However, when the population was grouped on the basis of countries or host plants, most of the genetic variation occurred within population (49.19–49.84%) as well as among the countries (48.56%) or host plants (49.01%), with much smaller amounts occurring among population.

**Table 6:** Result of AMOVA analysis of *Maruca vitrata* populations from nine countries in South- and Southeast Asia as well as sub-Saharan Africa, based on *coxI* sequence data.

Source of variation	df	Sum of squares	Variance components	Percentage of variation	Fixation indices
Among countries	8	310.25	0.5035**	48.56	Φ_CT_ = 0.4856
Among populations within countries	11	10.33	0.0166*	1.60	Φ_SC_ = 0.0310
Within all populations	666	344.17	0.5168**	49.84	Φ_ST_ = 0.5016
Total	685	664.75	1.0368		

* significant at *P* < 0.05

** highly significant at *P* < 0.01

**Table 7:** Result of AMOVA analysis of *Maruca* populations from four selected continents / regions based on *coxI* sequence data.

Source of variation	df	Sum of squares	Variance components	Percentage of variation	Fixation indices
Among continents / regions	3	2485.78	4.9343**	75.47	Φ_CT_ = 0.7547
Among populations within continents / regions	5	224.99	0.6261**	9.58	Φ_SC_ = 0.3905
Within all populations	810	791.64	0.9773**	14.95	Φ_ST_ = 0.8505
Total	818	3502.42	6.5378		

* significant at *P* < 0.05

** highly significant at *P* < 0.01

**Table 8:** Result of AMOVA analysis of *Maruca vitrata* populations from 22 host plants in South- and Southeast Asia as well as sub-Saharan Africa, based on *coxI* sequence data.

Source of variation	df	Sum of squares	Variance components	Percentage of variation	Fixation indices
Among host plant populations	8	310.25	0.5081**	49.01	Φ_CT_ = 0.4901
Among host plant populations within countries	40	29.63	0.0187**	1.80	Φ_SC_ = 0.0354
Within all populations	637	324.87	0.5100**	49.19	Φ_ST_ = 0.5081
Total	685	664.75	1.0368		

* significant at *P* < 0.05

** highly significant at *P* < 0.01

**Fig 1:**
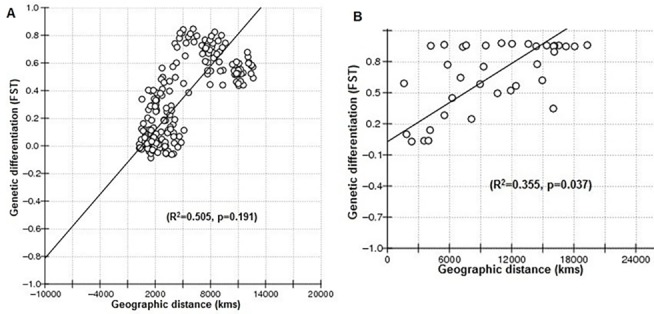
Genetic isolation-by-distance analysis by regression of genetic differentiation (*FST*) vs. geographic distance (km) (A) among *Maruca vitrata* population from different countries in Asia and Africa, and (B) among *Maruca* population from different continents or larger geographical regions.

When Mantel test was performed for country-based *M*. *vitrata* population in the current study, the results revealed no correlation between genetic differentiation (*F*_*ST*_) and geographical distance (R^2^ = 0.505, p = 0.191) (Fig 1A). For instance, the geographical distance between Benin (Central) and Malaysia (East) is 12,649 km, but the genetic differentiation (*F*_*ST*_) is 0.49. However, the geographical distance between India (Jharkhand, JK) and Bangladesh is 558 km only, whereas the genetic differentiation (*F*_*ST*_) is 0.34. Hence there is no detectable isolation by distance for the Asia and Africa *M*. *vitrata* population. However, the results of Mantel test for continents / larger geographical regions revealed the presence of a significant correlation between the genetic differentiation among the *Maruca* populations from Asia, Africa, Oceania and Latin America, and the geographical distance (R^2^ = 0.355, p = 0.037) (Fig 1B).

### Phylogenetic pattern

**Fig 2:**
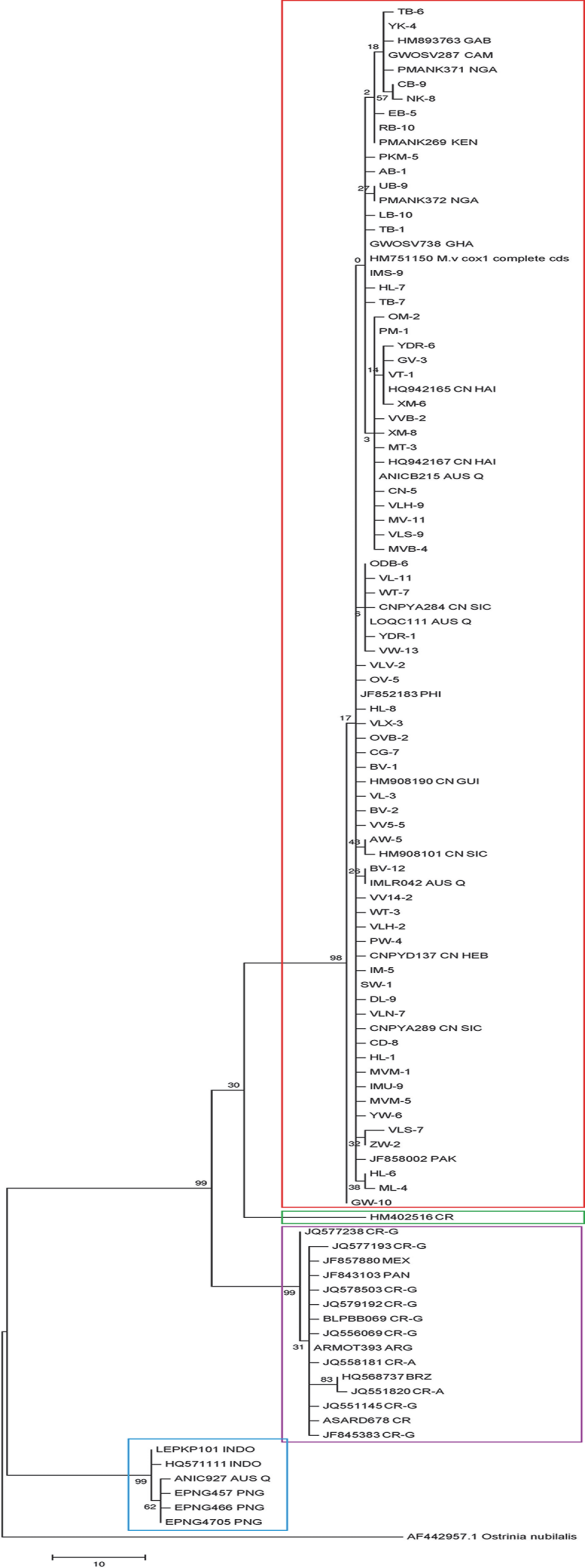
Phylogenetic relationship among *Maruca* sp. based upon a 615 bp mitochondrial coxI gene fragments using maximum parsimony (MP) algorithm. Countries abbreviated for the reference population in the phylogenetic tree are as follows: CAM—Cameroon; NGA—Nigeria; GAB—Gabon; KEN—Kenya; GHA—Ghana; AUS—Australia; PAK—Pakistan; CN—China; PHI—Philippines; CR—Costa Rica; BRZ—Brazil; MEX—Mexico; ARG—Argentina; PAN—Panama; INDO—Indonesia; PNG—Papua New Guinea. Groups are marked as below: Red—Group 1; Blue—Group 2; Purple—Group 3; Green—Group 4. Refer to Table 1 for the *M*. *vitrata* population details used in this study.

The intraspecific phylogenetic relationships based on the *cox1* sequences of *M*. *vitrata* are shown in Fig 2. Phylogenetic analysis based on partial *cox1* sequences was used to classify *M*. *vitrata* collected from different crops in different locations to species level. According to the maximum parsimony phylogenetic tree in the current study, the *M*. *vitrata* population from target countries was categorized into a single group (Group 1). The reference *M*. *vitrata* population from Africa (Cameroon, Gabon, Ghana, Kenya, Nigeria), Asia (China, Pakistan, Philippines) and part of Australia also aligned with our study population from South and Southeast Asia and sub-Saharan Africa. However, Group 2 was mainly comprised of reference *M*. *vitrata* population from Indonesia (Kalimantan), Australia and Papua New Guinea. Similarly, Group 3 included all the reference population from Latin America, except one sample from Costa Rica (HM402516) that formed a separate group (Group 4). Similar results were obtained with the maximum likelihood and neighbor-joining phylogenetic trees (tree not shown). All the sampled *M*. *vitrata* population formed a unique group, which is quite distinct from the *Maruca* population from Latin America and part of Oceania including Indonesia (Kalimantan). Thus, *M*. *vitrata* population from Latin America formed a paraphyletic group (Group 3). Although they are not as distant as the Oceania group (Group 2) from Group 1, the Latin American population formed a separate clade (Group 3).

### Automatic Barcoding Gap Discovery

**Fig 3:**
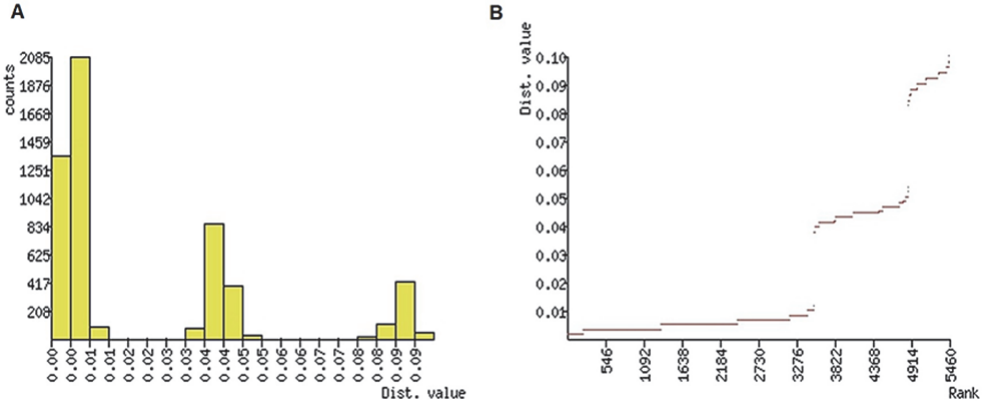
ABGD analysis- Distribution of *Maruca* spp. population K2P mean divergence in (A) histogram of distances, and (B) ranked distances.

**Fig 4:**
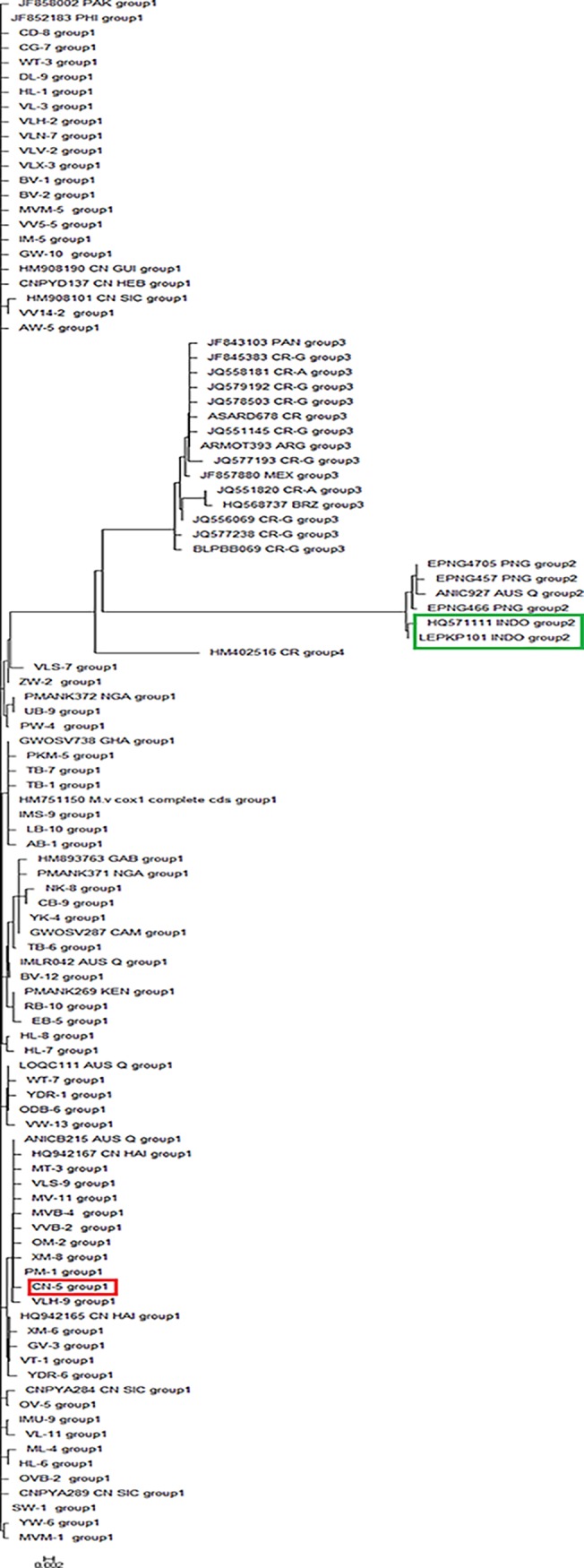
ABGD analysis- K2P divergence based Neighbor-joining tree based on *coxI* haplotypes. CN5 marked in red box is the population collected from Central Java, Indonesia, whereas two populations in green box (HQ571111 and LEPKP101) have originated from Kalimantan, Indonesia.

ABGD analysis resulted in four partitions with a prior of intraspecific divergence up to 0.008 (Fig 3A and 3B). Hence, ABGD tree also clustered the *coxI* sequences into four groups (putative species) (Fig 4), which is congruent with the phylogenetic tree. All the sampled *M*. *vitrata* population from Asia and Africa formed a unique group, and it is distinct from the *Maruca* population from Latin America and part of Oceania. Interestingly, one population from Costa Rica (HM402516) formed a separate group, similar to the phylogenetic tree. After constructing the ABGD tree, the samples were analyzed for F statistics and molecular variance based on ABGD tree grouping to validate the earlier results. This result is also in agreement with the earlier F statistics analysis based on country or continent based *Maruca* grouping (data not shown). AMOVA analysis was also performed with the *Maruca* population grouped in ABGD tree, and revealed that most of the genetic variation occurred among *Maruca* population from different ABGD Groups (91.85%) (data not shown).

### Haplotype network

**Fig 5:**
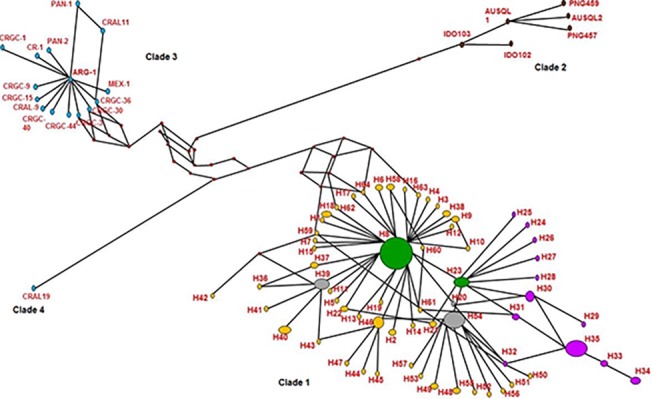
Median-joining haplotype network of the *cox1* gene of *Maruca* spp. in Asia, Africa, Oceania and Latin America. *cox1* haplotypes found in the study were included in the network analysis. Haplotype frequency is represented by the size of each node and red nodes represent hypothetical haplotypes (or median vectors). Asian haplotypes were marked in yellow nodes, African haplotypes in purple nodes, Latin American samples in blue nodes, and Oceania haplotypes in brown nodes. Nodes (H8 and H23) containing African, Asian and Australian haplotypes were marked in green, whereas nodes containing Asian and Australian haplotypes were marked in grey. Refer to Table 1 for the haplotype numbers and their corresponding *M*. *vitrata* population details used in this study. Countries abbreviated in this figure are as follows: AUSQL—Australia (Queensland); CRAL—Costa Rica (Alajuela); CRGC—Costa Rica (Guanacaste); MEX—Mexico; ARG—Argentina; PAN—Panama; IDO—Indonesia (Kalimantan); PNG—Papua New Guinea.

The haplotype network analysis involving the active *Maruca* haplotypes in the current study also revealed four distinct *Maruca* species (Fig 5). However, our study population from South and Southeast Asia and sub-Saharan Africa constituted the same clade. The radial expansion that occurred in the Asian population (highlighted in yellow in Fig 5), especially in Southeast Asia, with the largest haplotype (H8) as the founder node may indicate the potential geographical origin of *M*. *vitrata* population. The population then could have spread to sub-Saharan Africa (highlighted in purple in Fig 5, with H23 as the founder node). Most of the Papua New Guinea, and a few Australian and Indonesian (Kalimantan) *Maruca* population formed the second clade (highlighted in brown in Fig 5); they were very distant from the Asian—African clade and the Latin American clade. Although the Latin American *Maruca* population formed another major clade (clade 3, highlighted in blue in Fig 5, with ARG-1 as the founder node), only one *Maruca* population from Costa Rica formed a separate clade (clade 4). The Latin American clades were genetically distant from our Asian and African population. These results were basically congruent with the topology of the phylogenetic and ABGD trees, indicating distribution of genetic variation due to geographical separation [42].

## Discussion

An earlier study demonstrated that the DNA barcoding of the mitochondrial *coxl* gene is a viable method for determining molecular diversity and global distribution of *M*. *vitrata* mitochondrial haplotypes [17]. Although it included *M*. *vitrata* population from East Asia, West Africa, Australia and Puerto Rico, it had excluded Southeast Asia, the most probable center of origin for the genus *Maruca*. Our study has included extensive sampling of *M*. *vitrata* population from most of its host plants in South and Southeast Asia and compared them with the population collected from East and West Africa. Despite a broad geographic distribution among samples in the current study, all the samples fall within a single clade (Group 1), distinct from *Maruca* population from Oceania and Latin America. This is consistent with our morphological characterization of adult *Maruca* specimens from Benin, Kenya, Lao PDR, Taiwan, Thailand and Vietnam, in which the insects did not differ in their wing venation and the external genitalia structures of *M*. *vitrata*. Hence, the *Maruca* species infesting food legumes in these countries were confirmed as *M*. *vitrata* [43]. *M*. *vitrata* population from Asia and Africa seems to be genetically similar. An earlier study by Margam et al. [17] also confirmed that *M*. *vitrata* population from different regions including West Africa (Niger, Nigeria, and Burkina Faso), Taiwan and Australia formed the single clade in NJ and ME phylogenetic trees. However, it has to be noted that even subspecies could be sharply genetically differentiated and that *F*_*ST*_ values must be at least 0.25–0.30 for subspecies, or races, to be recognized [44–46]. Hence, the *M*. *vitrata* populations in Africa and Indonesia (Central Java) could be two different putative subspecies in the current study.

In our study, the *M*. *vitrata* population may have experienced a recent population expansion, indicated by the negative Tajima’s *D* and Fu’s *F*_*S*_ values for the overall population. Negative values of Tajima’s *D* are associated with selective sweeps or population expansion after a recent sharp decline [31]. Similarly, negative values of Fu’s *F*_*S*_ are usually caused by an excess of singletons in a population expansion event [47,48]. Hence, *M*. *vitrata* population in the target countries in our study could have experienced recent demographic expansion events. The statistically non-significant numbers indicating recent population growth could have been confined mostly by local geographical regions, except in Lao PDR [49], where demographic expansion may not be confined to that country alone, as it is relatively small and land-locked. Although *M*. *vitrata* population are speculated to expand locally, a large stable population with a long evolutionary history might be the case in Vietnam and Thailand which showed high haplotype and nucleotide diversities [42,50]. Similarly, Benin might have a stable population, whereas it was not the case for Kenya population. Thus, the population in Thailand, Vietnam and Benin seem to have attained some stability. Although these populations did not show any phenotypic variations based on the characterization of adult *M*. *vitrata* specimens, earlier studies documented the different responses of *M*. *vitrata* male moths to the same pheromone blends in West Africa including Benin [22], India [21], Taiwan [23], Thailand and Vietnam [51]. Hence, it is possible to speculate about the presence of two different subspecies in South- and Southeast Asia and in sub-Saharan Africa.

Our results from phylogenetic analysis are similar to the earlier results from Margam et al. [17], who showed that the *M*. *vitrata* population from Puerto Rico formed a separate clade in NJ and ME phylogenetic trees, and had a higher genetic distance compared to clade 1 (containing West African, Australian and Taiwanese population) and clade 2 (containing West African population). However, Margam et al. [17] included population from only one country in Latin America in their study, and did not include any population from Papua New Guinea and Indonesia. We included *M*. *vitrata* population from Argentina, Brazil, Costa Rica, Mexico and Panama as reference sequences. Although *M*. *vitrata* population from these Latin American countries formed a separate clade, they are closer to our study population than the Oceania *M*. *vitrata* population, which mainly came from Papua New Guinea and also from Australia (Queensland) and Indonesia (Kalimantan). It is interesting to note that Group 1, which contains all our study population including Indonesia (Central Java), with reference population from different provinces of China, Pakistan and the Philippines, also contains *M*. *vitrata* population from Australia. From this study, both Indonesia and Australia have two putative species of *Maruca* in total, and this observation was also supported by Herbison-Evans et al. [18], who reported two forms of *M*. *vitrata* in Australia. Our study confirmed that Indonesia also has two putative species of *Maruca*, as already documented in Australia. It also should be mentioned that one Papua New Guinea sample (JX970344) from the NCBI GenBank grouped with our study population, although we did not include it in our phylogenetic analysis because of its shorter length (589 bp). Hence, it is possible that Australia, Indonesia and Papua New Guinea may have two putative species of *Maruca*. The Indonesian reference population of *M*. *vitrata* that aligned with a part of Australia and Papua New Guinea population was collected from east Kalimantan (1.414 N, 115.976 E), whereas our study population from Indonesia that aligned with Southeast Asian and African population was collected in Magelang (7.467 S 110.217 E), central Java. Apparently these two populations are two different putative species based on the phylogenetic as well as ABGD analysis and *F*_*ST*_ values.

Compared with the Asian and African populations in the current study, the *Maruca* population in Papua New Guinea and Latin America could be two different putative species of *Maruca* based on the *F*_*ST*_ values. The *Ostrinia* species complex is a model for speciation of Lepidoptera and used as a basis for comparison of *M*. *vitrata* population by Margam et al. [17]. An overall nucleotide diversity of 0.0259 among *M*. *vitrata cox1* sequences [17] was similar to the range of 0.0015 to 0.0723 estimated among *Ostrinia* spp. using *cox1* sequence data [52,53], but higher than the 0.0130 shown between *O*. *nubilalis* and *O*. *furnacalis* mitochondrial genomes [54]. They also found that the level of mitochondrial variation among *M*. *vitrata* sequences was higher than previously reported species of *O*. *furnacalis* [55] or *O*. *nubilalis* (0.005–0.008) [56]. Because of the molecular variation within *M*. *vitrata cox1* samples, Margam et al. [17] suggested the existence of a species complex; in the current study, the overall nucleotide diversity among *Maruca cox1* sequences from different continents or larger geographical regions was similar to the value between *O*. *nubilalis* and *O*. *furnacalis* mitochondrial genomes. Hence, the three-fold higher nucleotide diversity for the Oceania *Maruca* population also suggests that it could be a different putative species.

Thus, it is reasonable to infer that Australia, Indonesia and Papua New Guinea have two putative species based on the genetic differentiation among the *Maruca* population, nucleotide diversity, and phylogenetic analysis. Our study population from Asia and Africa is also composed of two putative subspecies. Finally, both Oceania, especially Papua New Guinea and Latin America, have different putative species of *Maruca*. These findings are further supported by the species delineation method, ABGD in the current study. However, the *Maruca* adult specimens from Papua New Guinea and Latin America should be validated by characterizing the wing venation and genitalia characters for the presence of different *Maruca* species in future studies to establish the exact identity of those *Maruca* species.

Existence of two different putative species of *Maruca* in Australia, Indonesia, and Papua New Guinea strengthens the center of origin hypothesis for *Maruca* spp., since the probable center of origin is considered to be Southeast Asia. The Indo-Malaysian region is known to have other species of *Maruca*, such as *M*. *amboinalis* and *M*. *nigroapicalis* [14–16]. Besides other species of *Maruca*, this region is also reported to have several species-specific parasitoids including *Therophilus marucae* [57,58]. Thus, it is plausible to hypothesize that *Maruca* could have originated in Southeast Asia, especially in the region covering Vietnam, east Malaysia and parts of Indonesia, such as Kalimantan. The population then could have spread to sub-Saharan Africa, which was clearly demonstrated in the haplotype network in which the largest haplotype that contains *M*. *vitrata* from all the target countries in Asia and Africa served as the founder node.

Although Asia and Africa are two different continents, the continuous land area and the availability of host plants year-round could have favored the migration of *Maruca*. It has to be noted that *Maruca* is a strong flier and could arrive in large numbers. Thus the genetic difference in the *Maruca* populations of Asia and Africa is not so significant, although the geographical distance is higher, as evidenced in the isolation by distance model used in this study. However, the *M*. *vitrata* population within Africa is slowly evolving to be a different putative subspecies. For instance, alternation of the flowering pattern of a number of wild and cultivated host plants on a south-north gradient was found to influence the migration of this insect from coastal areas to dry savannas in West Africa [59]. During this migration, *M*. *vitrata* chooses the most favorable host plants and conditions and thus increases its population exponentially in each new generation. Such population growth is confined to local regions alone, as supported by non-significant but negative Tajima’s *D* value for the African *Maruca* population in the current study. Confinement will maintain the gene flow within local regions, which may enable the African population to evolve a separate lineage from the Asian *M*. *vitrata* population. Besides *coxI*, the phylogenetic analysis based on the arrestin gene from *M*. *vitrata* (MaviArr2) also confirmed the grouping of the African population in a separate clade from the Asian population [60]. Our preliminary phylogenetic analysis based on internal transcribed spacer 2 (ITS2) region also complemented the diversity analysis of *M*. *vitrata* population from Asia and Africa based on *coxI* gene sequences [61]. This could be a possible reason why Asian and African *M*. *vitrata* populations responded differently to the same blends of pheromone lures. However, this puzzle may not be resolved until we know the complete composition of pheromone blends produced and released by *M*. *vitrata* female moths in Asia and Africa, since the existing literature does not show any variation in pheromone composition. Hence, it is imperative to profile the complete list of components in the sex pheromone of *M*. *vitrata* populations occurring in Asia and Africa.

It is clearly evident that the *Maruca* populations in Papua New Guinea and Latin America are two different putative species, which are quite different from our *M*. *vitrata* population in Asia and Africa. One possible reason for the difference is that these populations are geographically isolated, which was also demonstrated in the isolation by distance model. Since Latin America is quite far from other countries or regions, it is highly unlikely that a Latin American *Maruca* population could reach Asia or Africa, despite the species’ strong flying ability. Secondly, *Maruca* has several host plants including green beans and soybean, which are cultivated on larger acreage in Latin America. Hence, it is possible that this species of *Maruca* has been prevalent only in Latin American countries. Finally, we do not have any information on the sex pheromones of this *Maruca* population in Latin America. Hence, insights on pheromones of *Maruca* from this region will also be useful to understand the *Maruca* species complex in the future.

Unlike Latin American *Maruca* species, the species from Papua New Guinea is restricted neither to this country nor the Pacific Islands alone. This species is also present in Indonesia (Kalimantan) and Australia, where *M*. *vitrata* is a predominant species. Although Papua New Guinea is a group of islands, migration of *Maruca* from Papua New Guinea to Australia or Indonesia (Kalimantan), or *vice versa*, cannot be ruled out. The haplotype network showed that the founder node for this *Maruca* species in Australia, Indonesia (Kalimantan) and Papua New Guinea is the Indonesian haplotype. Hence, this species could have originated in Kalimantan region and spread to the Pacific Islands, where it adapted to local conditions. Sampling additional *Maruca* populations from this region could offer more insight into the species’ migration patterns. In addition, both Tajima’s *D* and Fu’s *F*_*S*_ values are positive for Oceania. Positive values of Tajima’s *D* are associated with balancing selection where the frequency of polymorphisms is equal [31]. Positive values of Fu’s *F*_*S*_ indicate a deficiency of singletons, which is expected from a recent population decline [47,48]. Hence, a recent population expansion may not be the case for the Oceania *Maruca* population because of the smaller geographical areas in the Pacific island countries and limited availability of host plants. For instance, beans and other legumes do not make up much of the diet in Papua New Guinea and thus are not widely grown, which is not the case in several other tropical countries. Very high haplotype and nucleotide diversities for the Oceania *Maruca* population also confirm a large stable population with a long evolutionary history in this region, especially in Papua New Guinea [42,50].

Finally, the current study did not differentiate between the *M*. *vitrata* populations among host plants within countries. This is consistent with a recent finding from Benin, where the *M*. *vitrata* population were compared between cultivated host plant (cowpea) and three alternative host plants [62], and concluded that host plants do not significantly influence the genetic structure of *M*. *vitrata*. However, their study involved population from three divisions in southern Benin only. In contrast, our study involved several populations collected from different countries in South and Southeast Asia as well as sub-Saharan Africa. Despite wider geographical sampling involving more than 20 host plants, we are unable to establish any genetic differences associated with host plants.

## Conclusions

The *cox1* gene has been used to understand the phylogenetic relationship of geographically different *M*. *vitrata* population, but there are no previous studies that included *M*. *vitrata* population from Southeast Asia, the probable center of origin for *Maruca*, or population from East Africa. We have done extensive sampling from different host plant species in South- and Southeast Asia as well as East and West Africa. We did not identify any population separation based on host plants. However, our results based on *cox1* confirmed that the *M*. *vitrata* has different putative subspecies in Asia and sub-Saharan Africa, although they cannot be differentiated based on their morphological characters. This is possible because of recent local population expansions and accumulated mutations in the silent sites, which are supported by the negative Tajima’s *D* and Fu’s *F*_*S*_ values in our study. However, the *Maruca* population in Latin America and Oceania, especially Papua New Guinea, seems to be a different species, based on the extremely high *F*_*ST*_ values obtained, phylogenetic and ABGD trees. In addition, two putative species of *Maruca* seem to occur in Australia, Indonesia and Papua New Guinea. This observation needs further validation through morphological characterization based on wing venation and genitalia characters. The genetic differences in *Maruca* population, especially the distinct population in Asia, sub-Saharan Africa, Latin America and Oceania, should be carefully considered when designing integrated pest management strategies, especially those based on sex pheromones.
